# Analysis of Risk Factors and Construction of a Prediction Model for Preoperative Concurrent Pathogenic Pyuria in Patients With Ureteral Calculi

**DOI:** 10.1093/ofid/ofaf733

**Published:** 2025-12-11

**Authors:** Hengjiang Wu, Wei Zheng, Huayu Bao, Yuandi Fu, Chunhua Lin, Zhiguang Liao, Yijun Ye, Mengzhao Wei, Yu Zhang

**Affiliations:** Department of Urology, The First People's Hospital of Qinzhou, Qinzhou, Guangxi Zhuang Autonomous Region, People's Republic of China; Department of Urology, The First People's Hospital of Qinzhou, Qinzhou, Guangxi Zhuang Autonomous Region, People's Republic of China; Department of Urology, The First People's Hospital of Qinzhou, Qinzhou, Guangxi Zhuang Autonomous Region, People's Republic of China; Department of Urology, The First People's Hospital of Qinzhou, Qinzhou, Guangxi Zhuang Autonomous Region, People's Republic of China; Department of Urology, The First People's Hospital of Qinzhou, Qinzhou, Guangxi Zhuang Autonomous Region, People's Republic of China; Department of Urology, The First People's Hospital of Qinzhou, Qinzhou, Guangxi Zhuang Autonomous Region, People's Republic of China; Department of Urology, The First People's Hospital of Qinzhou, Qinzhou, Guangxi Zhuang Autonomous Region, People's Republic of China; Department of Urology, The First People's Hospital of Qinzhou, Qinzhou, Guangxi Zhuang Autonomous Region, People's Republic of China; Department of Scientific Research, The First People's Hospital of Qinzhou, Qinzhou, Guangxi Zhuang Autonomous Region, People's Republic of China

**Keywords:** hydronephrosis, pathogenic, preoperative, pyuria, ureteral calculi (UC)

## Abstract

**Background:**

This study aims to analyze the risk factors for preoperative pyuria in patients with ureteral calculi (UC). On the basis of clarifying the presence of pyuria, it focuses on exploring the risk factors for concurrent pathogenic pyuria, and meanwhile constructs a simple and practical prediction model to assess the risk of its occurrence, so as to provide a reference basis for clinical early preoperative intervention in such patients.

**Methods:**

From January 2019 to May 2025, a total of 1182 patients with UC were enrolled in this study. First, they were divided into a pyuria group and a nonpyuria group. Logistic regression analyses were used to screen out the risk factors associated with pyuria. To enhance the reliability and robustness of the results, a sensitivity analysis was further performed to verify the conclusions. Subsequently, the pyuria group was further divided into 2 subgroups, namely a pathogenic pyuria group and a sterile pyuria group. Using the same methods mentioned above, the relevant risk factors for preoperative concurrent pathogenic pyuria in patients with UC were identified, and a corresponding prediction model was constructed, which was then validated using receiver operating characteristic (ROC) curves and calibration curves.

**Results:**

A total of 1182 patients with UC were enrolled in this study, including 250 cases (21.15%) receiving nonsurgical treatment and 932 cases (78.85%) undergoing surgical treatment. Among them, 630 cases (53.30%) were diagnosed with pyuria. Multivariate logistic regression analysis combined with sensitivity analysis showed that female gender (*P* = .005), bilateral UC (*P* = .007), stone size (*P* < .05), and hydronephrosis size (*P* < .05) were independent risk factors for preoperative pyuria in such patients. A further subgroup analysis was performed on 630 patients with pyuria, including 135 cases (21.43%) of pathogenic pyuria and 495 cases (78.57%) of sterile pyuria. Analysis using the same methods mentioned above showed that female gender (*P* < .001), comorbid diabetes mellitus (*P* = .032), and hydronephrosis size > 40 mm (*P* = .022) were independent risk factors for preoperative concurrent pathogenic pyuria in patients with UC. A prediction model was constructed by combining the above indicators with urinary white blood cells > 60 per high-power field as predictive variables. The area under the ROC curve of this model was 0.764, indicating good predictive ability; the Hosmer–Lemeshow test (*P* = .989) showed good goodness of fit; and the calibration curve demonstrated good consistency of the model.

**Conclusions:**

This study not only confirms that female gender, bilateral UC, stone size, and hydronephrosis size are independent risk factors for preoperative pyuria in patients with UC, but also further identifies that female gender, comorbid diabetes mellitus, and hydronephrosis size > 40 mm are independent risk factors for preoperative concurrent pathogenic pyuria in such patients. The prediction model constructed accordingly can effectively assess the risk of preoperative concurrent pathogenic pyuria in female patients with UC, providing a practical reference basis for clinicians to implement early preoperative intervention in such patients.

As one of the common types of urolithiasis, UC are experiencing a steady increase in both incidence and prevalence [1]. UC are prone to concurrent urinary tract infections (UTIs); severe infections can progress to urosepsis, septic shock, or even be life-threatening [2–4]. Some patients with UC can expel the stones spontaneously through conservative treatment, while others require surgical intervention. It should be particularly noted that uncontrolled UTI is a contraindication to such surgery [5]. Currently, the clinical diagnosis of UTIs typically employs urinalysis and urine culture methods [6]. Whether anti-infective treatment is required before UC surgery mainly depends on the presence of infection, especially UTI. Urinalysis can quickly determine the presence of preoperative pyuria, while confirming the presence of urinary tract pathogenic infection requires urine culture, whose results take at least 48 hours or even longer. If urine culture indicates concurrent urinary tract pathogenic infection, the patient must first receive anti-infective treatment and undergo repeated urine cultures until the results are negative before surgery can be performed. This will undoubtedly significantly prolong the perioperative period and increase the economic burden. Therefore, timely identification of UC patients with concurrent pathogenic pyuria and implementation of early preoperative intervention are of great significance for shortening the length of hospital stay and optimizing the diagnosis and treatment process.

Urosepsis is a common complication after UC surgery, and it is particularly more prevalent in patients with positive preoperative urine culture results [7, 8]. Previous studies have shown that early use of antibiotics for anti-infective treatment is closely associated with patients’ prognosis, and this is particularly significant in patients with severe infections [9–11]. Therefore, for patients with UC who have concurrent pathogenic pyuria and require surgical treatment, early preoperative anti-infective therapy is crucial. Given the delayed nature of urine culture results, the early identification of such patients and the implementation of early preoperative interventions remain a challenge in clinical practice. Currently, most existing studies have focused on postoperative infections in patients with UC [12–15]. We have not yet retrieved any relevant studies on preoperative pyuria and concurrent pathogenic pyuria in patients with UC in PubMed; thus, this study can be regarded as the first exploration into this issue. To address this, this study will, on the one hand, explore the risk factors for preoperative pyuria in patients with UC, and on the other hand, focus on analyzing the risk factors for preoperative concurrent pathogenic pyuria in such patients and constructing a targeted prediction model. It aims to achieve early identification of UC patients with preoperative concurrent pathogenic pyuria and provide a basis for clinicians to implement early preoperative interventions.

## METHODS

### Study Population

This study was approved by the Medical Ethics Committee of The First Peoples Hospital of Qinzhou (Approval No.: 2 021 082). Due to the retrospective nature of the study, the Medical Ethics Committee of The First People's Hospital of Qinzhou waived the need for obtaining informed consent. All procedures in this study were performed in accordance with the Declaration of Helsinki. This study enrolled 1182 participants from January 2019 to May 2025. To avoid interfering with the results of urinalysis and etiological culture, all participants underwent urinalysis and urine culture examinations after admission but prior to any medical intervention, and none had a history of antibiotic use before enrollment. Among them, there were 250 nonsurgical cases and 932 surgical cases, and 630 of these participants were diagnosed with pyuria. The participants were divided into a pyuria group and a nonpyuria group according to the diagnostic criteria for pyuria [16, 17], with the inclusion criteria for each group as follows: Pyuria group: (1) diagnosed with UC; (2) microscopic examination of urine showing ≥5 white blood cells (WBC)/high-power field (HPF); (3) no history of urinary system invasive procedures in the past 3 months. Nonpyuria group: (1) diagnosed with UC; (2) microscopic examination of urine showing <5 WBC/HPF; (3) no history of urinary system invasive procedures in the past 3 months. Exclusion criteria: (1) Presence of any malignant tumor; (2) Severe infections in other body sites; (3) Complicated with lower urinary tract calculi; (4) History of urinary system invasive procedures in the past 3 months.

A subgroup analysis was performed on 630 patients with pyuria, including 106 nonsurgical cases and 524 surgical cases; among them, 135 cases had bacteria or fungi detected in urine culture (including 8 cases of fungal infection and 127 cases of bacterial infection). Based on this, the subjects were further divided into a pathogenic pyuria group and a sterile pyuria group for subsequent analysis.

### Data Collection

The clinical data of patients were collected, including the following indicators: (1) Demographic data: sex, age, body mass index (BMI), diabetes mellitus, and history of hypertension; (2) laboratory tests: (i) Serological tests: serum creatinine; (ii) Urine tests: microscopic examination of urinary WBC, urine culture, urine was mainly collected from the bladder, with clean midstream urine obtained directly with self-urination; (3) Imaging examinations: side of the stone, stone location, number of stones (single/multiple), maximum stone diameter, maximum diameter of hydronephrosis, presence of concurrent renal calculi, etc.

### Statistical Analysis

A total of 12 indicators were collected as research variables in this study. Among them, a serum creatinine level of ≥106 μmol/L were defined as impaired renal function, and <106 μmol/L as normal renal function. Categorical variables were presented as counts (percentages), with intergroup comparisons performed using the χ^2^squared test. Independent risk factors were identified via multivariate logistic regression analysis. After excluding nonsurgical patients, sensitivity analysis was conducted on surgical patients to ensure the robustness of the results.

On the basis of diagnosing pyuria, further subgroup analysis was conducted. Using the same analytical method mentioned above, independent predictive indicators were screened out. These indicators, together with urine WBC >60/HPF, were used as predictive variables to construct a prediction model, so as to predict the occurrence of preoperative concurrent pathogenic pyuria in patients with UC.

The accuracy of the model was evaluated using the area under the curve (AUC): an AUC in the range of 0.5–0.7 indicates low accuracy, 0.7–0.9 indicates acceptable accuracy, and >0.9 indicates excellent accuracy [2]. The Hosmer-Lemeshow test was used to verify the goodness of fit of the model, calibration curves were utilized to analyze the differences between predicted probabilities and actual observed values, and internal validation was completed via 1000 repeated bootstrap samples. Data analysis and graphing were performed using SPSS 27.0 software and Statistical Platform for Life Sciences, with a *P* < .05 considered statistically significant for differences.

## RESULTS

### Patients’ Demographic Characteristics and Clinical Data

A total of 1182 participants were enrolled in this study, including nonsurgical patients (n = 250, 21.15%) and surgical patients (n = 932, 78.85%). All participants were divided into 2 groups based on the presence of pyuria: the pyuria group (n = 630, 53.30%) and the nonpyuria group (n = 552, 46.70%). The results of intergroup comparison showed that there were statistically significant differences between the 2 groups in terms of sex, concomitant kidney stones, UC (unilateral/bilateral), stone size, hydronephrosis size, and stone location (all *P* < .05), while no statistically significant differences were found in other indicators (all *P* > .05). Details are shown in Table 1.

**Table 1.** Patients’ Demographic Characteristics and Clinical Data

Variables	All (n = 1182)	Nonpyuria Group (n = 552)	Pyuria Group (n = 630)	χ^2^	*P*
**Sex**				10.334	.001^a^
Male	700	354 (50.6%）	346 (49.4%）	…	
Female	482	198（41.1%）	284（58.9%）	…	
**Age (y)**				.638	.727
18–35	176	87（49.4%）	89（50.6%）	…	
36–50	398	185（46.5%）	213（53.5%）	…	
＞50	608	280（46.1%）	328（53.9%）	…	
**BMI**				2.668	.102
＜28	1061	487（45.9%）	574（54.1%）	…	
≥28	121	65（53.7%）	56（46.3%）	…	
**Diabetes mellitus**				.000	.994
NO	1090	509（46.7%）	581（53.3%）	…	
Yes	92	43（46.7%）	49（53.3%）	…	
**Hypertension**				1.143	.285
NO	926	440（47.5%）	486（52.5%）	…	
Yes	256	112（43.8%）	144（56.2%）	…	
**Concomitant kidney stones**				8.021	.046^a^
No concomitant kidney stones	400	200（50.0%）	200（50.0%）	…	
Concomitant ipsilateral kidney stones	303	152（50.2%）	151（49.8%）	…	
Concomitant contralateral kidney stones	162	66（40.7%）	96（59.3%）	…	
Concomitant bilateral kidney stones	317	134（42.3%）	183（57.7%）	…	
**UC(unilateral/bilateral)**				14.052	<.001^a^
Unilateral UC	1056	513（48.6%）	543（51.4%）	…	
Bilateral UC	126	39（31.0%）	87（69.0%）	…	
**Single/multiple stones**				.750	.387
Single stone	997	471（47.2%）	526（52.8%）	…	
Multiple stones	185	81（43.8%）	104（56.2%）	…	
**Stone size(mm)**				43.863	<.001^a^
≤6	337	207（61.4%）	130（38.6%）	…	
7–15	729	306（42.0%）	423（58.0%）	…	
＞15	116	39（33.6%）	77（66.4%）	…	
**Hydronephrosis size(mm)**				44.625	<.001^a^
≤10	165	114（69.1%）	51（30.9%）	…	
11–20	348	168（48.3%）	180（51.7%）	…	
21–30	338	139（41.1%）	199（58.9%）	…	
31–40	180	70（38.9%）	110（61.1%）	…	
＞40	151	61（40.4%）	90（59.6%）	…	
**Stone location**				14.107	.003^a^
Upper segment	688	298（43.3%）	390（56.7%）	…	
Middle segment	333	169（50.8%）	164（49.2%）	…	
Lower segment	109	65（59.6%）	44（40.4%）	…	
Multiple segment	52	20（38.5%）	32（61.5%）	…	
**Whether renal function impairment**				1.938	.164
NO	932	445（47.7%）	487（52.3%）	…	
Yes	250	107（42.8%）	143（57.2%）	…	

^a^Statistically significant. BMI, body mass index; UC, ureteral calculi.

### Logistic Regression Analysis and Risk Factor Identification

By sequentially conducting univariate and multivariate logistic regression analyses, the independent risk factors for pyuria in patients with UC were identified, with the results detailed in Table 2. The multivariate logistic regression analysis showed that female gender, bilateral UC, stone size, and hydronephrosis size were independent risk factors for pyuria in these patients.

**Table 2.** Risk Factors for Pyuria in Patients With UC

Variables	Univariable			Multivariable		
	OR	95% CI	P	OR	95% CI	*P*
**Sex**						
Male	…	…		…	…	…
Female	1.468	(1.161–1.855)	.001^a^	1.442	(1.118–1.858)	.005^a^
**Age (y)**						
18–35	…	…		…	…	…
36–50	1.125	(0.789–1.605)	.514	1.008	(0.692–1.468)	.967
50	1.145	(0.818–1.602)	.429	0.893	(0.615–1.295)	.550
**BMI**						
＜28	…	…		…	…	…
≥28	0.731	(0.501–1.066)	.103	0.705	(0.475–1.045)	.081
**Diabetes mellitus**						
NO	…	…		…	…	…
Yes	0.998	(0.652–1.529)	.994	0.987	(0.623–1.564)	.957
**Hypertension**						
NO	…	…		…	…	…
Yes	1.164	(0.881–1.538)	.285	1.128	(0.828–1.535)	.446
**Concomitant kidney stones**						
No concomitant kidney stones	…	…		…	…	…
Concomitant ipsilateral kidney stones	0.993	(0.737–1.339)	.965	0.814	(0.593–1.117)	.202
Concomitant contralateral kidney stones	1.455	(1.005–2.105)	.047^a^	1.266	(0.859–1.866)	.232
Concomitant bilateral kidney stones	1.366	(1.015–1.838)	.040^a^	1.027	(0.745–1.418)	.869
**UC(unilateral/bilateral)**						
Unilateral UC	…	…		…	…	…
Bilateral UC	2.108	(1.418–3.133)	<.001^a^	1.828	(1.187–2.816)	.006^a^
**Single/multiple stones**						
Single stone	…	…		…	…	…
Multiple stones	1.150	(0.838–1.577)	.387	0.930	(0.648–1.334)	.693
**Stone size(mm)**						
≤6	…	…		…	…	…
7–15	2.201	(1.690–2.866)	<.001^a^	1.671	(1.238–2.256)	<.001^a^
＞15	3.144	(2.018–4.897)	<.001^a^	2.348	(1.428–3.858)	<.001^a^
**Hydronephrosis size(mm)**						
≤10	…	…		…	…	…
11–20	2.395	(1.619–3.542)	<.001^a^	2.092	(1.374–3.186)	<.001^a^
21–30	3.200	(2.156–4.750)	<.001^a^	2.272	(1.469–3.514)	<0.001^a^
31–40	3.513	(2.249–5.486)	<.001^a^	2.327	(1.425–3.800)	<.001^a^
＞40	3.298	(2.075–5.242)	<.001^a^	2.078	(1.242–3.477)	.005^a^
**Stone location**						
Upper segment	…	…		…	…	…
Middle segment	0.741	(0.570–0.964)	.026^a^	0.946	(0.708–1.264)	.708
Lower segment	0.517	(0.343–0.780)	.002^a^	0.911	(0.574–1.446)	.693
Multiple segment	1.223	(0.685–2.181)	.496	1.414	(0.738–2.709)	.296
**Whether renal function impairment**						
NO	…	…		…	…	…
Yes	1.221	(0.922–1.618)	.164	1.060	(0.771–1.457)	.721

^a^Statistically significant. BMI, body mass index; UC, ureteral calculi.

### Sensitivity Analysis and Verification of Risk Factors

After excluding nonsurgical patients, this study conducted a sensitivity analysis on 932 surgical patients, sequentially performing univariate and multivariate logistic regression analyses, with the results detailed in Table 3. The results of the sensitivity analysis further confirmed that female gender, bilateral UC, stone size, and hydronephrosis size are independent risk factors for preoperative pyuria in patients with UC.

**Table 3.** Risk Factors for Preoperative Pyuria in Patients With UC(Sensitivity Analysis)

Variables	Univariable			Multivariable		
	OR	95% CI	P	OR	95% CI	*P*
**Sex**						
Male	…	…		…	…	…
Female	1.371	(1.056–1.781)	.018^a^	1.503	(1.131–1.998)	.005^a^
**Age (y)**						
18–35	…	…		…	…	…
36–50	1.154	(0.763–1.747)	.497	1.031	(0.666–1.594)	.892
＞50	1.157	(0.784–1.709)	.463	0.957	(0.623–1.471)	.842
**BMI**						
＜28	…	…		…	…	…
≥28	0.705	(0.461–1.078)	.107	0.705	(0.453–1.098)	.122
**Diabetes mellitus**						
NO	…	…		…	…	…
Yes	0.905	(0.565–1.449)	.677	0.960	(0.580–1.589)	.873
**Hypertension**						
NO	…	…		…	…	…
Yes	0.982	(0.721–1.336)	.905	0.944	(0.674–1.324)	.740
**Concomitant kidney stones**						
No concomitant kidney stones	…	…		…	…	…
Concomitant ipsilateral kidney stones	0.813	(0.576–1.147)	.238	0.711	(0.495–1.022)	.065
Concomitant contralateral kidney stones	1.293	(0.853–1.961)	.226	1.245	(0.808–1.918)	.320
Concomitant bilateral kidney stones	1.202	(0.857–1.687)	.287	1.002	(0.697–1.441)	.991
**UC(unilateral/bilateral)**						
Unilateral UC	…	…		…	…	…
Bilateral UC	2.048	(1.334–3.142)	.001^a^	1.914	(1.198–3.057)	.007^a^
**Single/multiple stones**						
Single stone	…	…		…	…	…
Multiple stones	1.145	(0.807–1.623)	.448	1.002	(0.677–1.482)	.992
**Stone size(mm)**						
≤6	…	…		…	…	…
7–15	1.768	(1.260–2.482)	<.001^a^	1.621	(1.126–2.333)	.009^a^
＞15	2.623	(1.590–4.325)	<.001^a^	2.426	(1.404–4.193)	.002^a^
**Hydronephrosis size(mm)**						
≤10	…	…		…	…	…
11–20	2.091	(1.222–3.576)	.007^a^	2.061	(1.152–3.686)	.015^a^
21–30	2.550	(1.512–4.298)	<.001^a^	2.226	(1.269–3.903)	.005^a^
31–40	2.721	(1.547–4.784)	<.001^a^	2.210	(1.206–4.050)	.010^a^
＞40	2.620	(1.468–4.675)	.001^a^	2.009	(1.072–3.767)	.030^a^
**Stone location**						
Upper segment	…	…		…	…	…
Middle segment	0.799	(0.596–1.071)	.134	0.930	(0.674–1.284)	.660
Lower segment	0.798	(0.443–1.439)	.454	0.998	(0.526–1.894)	.995
Multiple segment	1.469	(0.757–2.848)	.255	1.675	(0.800–3.507)	.171
**Whether renal function impairment**						
NO	…	…		…	…	…
Yes	1.278	(0.933–1.752)	.127	1.124	(0.787–1.605)	.522

^a^Statistically significant. BMI, body mass index; UC, ureteral calculi.

### Patients’ Demographic Characteristics and Clinical Data(Subgroup Analysis)

A total of 630 patients with confirmed pyuria were included in the subgroup analysis, with nonsurgical patients (n = 106, 16.83%) and surgical patients (n = 524, 83.17%). Among them, there were 135 (21.43%) cases in the pathogenic pyuria group and 495 (78.57%) cases in the sterile pyuria group. Intergroup comparison showed that there were statistically significant differences between the 2 groups in terms of sex, diabetes mellitus, and hydronephrosis size (all *P* < .05), while no statistically significant differences were found in other indicators (all *P* > .05). Details are shown in Table 4.

**Table 4.** Patients’ Demographic Characteristics and Clinical Data(Subgroup Analysis)

Variables	All (n = 630)	Sterile Pyuria Group (n = 495)	Pathogenic Pyuria Group (n = 135)	χ^2^	*P*
**Sex**				55.401	<.001^a^
Male	346	310 (89.6%）	36 (10.4%）	…	
Female	284	185（65.1%）	99（34.9%）	…	
**Age (y)**				5.734	.057
18–35	89	78（87.6%）	11（12.4%）	…	
36–50	213	168（78.9%）	45（21.1%）	…	
＞50	328	249（75.9%）	79（24.1%）	…	
**BMI**				2.910	.088
＜28	574	456（79.4%）	118（20.6%）	…	
≥28	56	39（69.6%）	17（30.4%）	…	
**Diabetes mellitus**				5.553	.018^a^
NO	581	463（79.7%）	118（20.3%）	…	
Yes	49	32（65.3%）	17（34.7%）	…	
**Hypertension**				0.184	.668
NO	486	380（78.2%）	106（21.8%）	…	
Yes	144	115（79.9%）	29（20.1%）	…	
**Concomitant kidney stones**				5.152	.161
No concomitant kidney stones	200	156（78.0%）	44（22.0%）	…	
Concomitant ipsilateral kidney stones	151	110（72.8%）	41（27.2%）	…	
Concomitant contralateral kidney stones	96	80（83.3%）	16（16.7%）	…	
Concomitant bilateral kidney stones	183	149（81.4%）	34（18.6%）	…	
**UC(unilateral/bilateral)**				3.495	.062
Unilateral UC	543	420（77.3%）	123（22.7%）	…	
Bilateral UC	87	75（86.2%）	12（13.8%）	…	
**Single/multiple stones**				0.201	.654
Single stone	526	415（78.9%）	111（21.1%）	…	
Multiple stones	104	80（76.9%）	24（23.1%）	…	
**Stone size(mm)**				1.729	.421
≤6	130	106（81.5%）	24（18.5%）	…	
7–15	423	326（77.1%）	97（22.9%）	…	
＞15	77	63（81.8%）	14（18.2%）	…	
**Hydronephrosis size(mm)**				10.368	.035^a^
≤10	51	47（92.2%）	4（7.80%）	…	
11–20	180	147（81.7%）	33（18.3%）	…	
21–30	199	155（77.9%）	44（22.1%）	…	
31–40	110	80（72.7%）	30（27.3%）	…	
＞40	90	66（73.3%）	24（26.7%）	…	
**Stone location**				2.265	.519
Upper segment	390	306（78.5%）	84（21.5%）	…	
Middle segment	164	125（76.2%）	39（23.8%）	…	
Lower segment	44	38（86.4%）	6（13.6%）	…	
Multiple segment	32	26（81.3%）	6（18.7%）	…	
**Whether renal function impairment**				3.139	.076
NO	487	375（77.0%）	112（23.0%）	…	
Yes	143	120（83.9%）	23（16.1%）	…	

^a^Statistically significant. BMI, body mass index; UC, ureteral calculi.

### Logistic Regression Analysis and Risk Factor Identification(Subgroup Analysis)

Univariate and multivariate logistic regression analyses were performed on 630 patients with pyuria, with the results detailed in Table 5. Multivariate analysis showed that female gender, comorbid diabetes mellitus, and hydronephrosis size > 30 mm were independent risk factors for concurrent pathogenic pyuria in patients with UC.

**Table 5.** Risk Factors for Concurrent Pathogenic Pyuria in Patients With UC(Subgroup Analysis)

Variables	Univariable			Multivariable		
	OR	95% CI	P	OR	95% CI	*P*
**Sex**						
Male	…	…		…	…	…
Female	4.608	(3.020–7.032)	<.001^a^	4.462	(2.834–7.027)	<.001^a^
**Age (y)**						
18–35	…	…		…	…	…
36–50	1.899	(0.932–3.870)	.077	1.786	(0.837–3.811)	.134
＞50	2.250	(1.140–4.441)	.019^a^	1.757	(0.834–3.698)	.138
**BMI**						
＜28	…	…		…	…	…
≥28	1.684	(0.920–3.083)	.091	1.937	(0.982–3.821)	.056
**Diabetes mellitus**						
NO	…	…		…	…	…
Yes	2.084	(1.119–3.883)	.021^a^	2.178	(1.075–4.414)	.031^a^
**Hypertension**						
NO	…	…		…	…	…
Yes	0.904	(0.570–1.433)	.668	0.638	(0.375–1.084)	.097
**Concomitant kidney stones**						
No concomitant kidney stones	…	…		…	…	…
Concomitant ipsilateral kidney stones	1.321	(0.809–2.158)	.265	1.395	(0.810–2.403)	.231
Concomitant contralateral kidney stones	0.709	(0.377–1.335)	.287	0.686	(0.343–1.369)	.285
Concomitant bilateral kidney stones	0.809	(0.490–1.335)	.407	0.702	(0.401–1.229)	.215
**UC(unilateral/bilateral)**						
Unilateral UC	…	…		…	…	…
Bilateral UC	0.546	(0.288–1.038)	.065	0.614	(0.294–1.279)	.193
**Single/multiple stones**						
Single stone	…	…		…	…	…
Multiple stones	1.122	(0.679–1.853)	.654	0.976	(0.524–1.816)	.938
**Stone size(mm)**						
≤6	…	…		…	…	…
7–15	1.314	(0.799–2.162)	.282	1.169	(0.657–2.078)	.596
＞15	0.981	(0.473–2.035)	.960	0.783	(0.332–1.846)	.576
**Hydronephrosis size(mm)**						
≤10	…	…		…	…	…
11–20	2.638	(0.888–7.833)	.081	1.921	(0.602–6.129)	.270
21–30	3.335	(1.139–9.766)	.028^a^	2.664	(0.837–8.475)	.097
31–40	4.406	(1.461–13.286)	.008^a^	3.355	(1.017–11.066)	.047^a^
＞40	4.273	(1.390–13.130)	.011^a^	5.241	(1.544–17.788)	.008^a^
**Stone location**						
Upper segment	…	…		…	…	…
Middle segment	1.137	(0.737–1.753)	.562	0.866	(0.529–1.419)	.569
Lower segment	0.575	(0.235–1.407)	.225	0.782	(0.286–2.138)	.631
Multiple segment	0.841	(0.335–2.109)	.712	0.667	(0.212–2.094)	.488
**Whether renal function impairment**						
NO	…	…		…	…	…
Yes	0.642	(0.392–1.051)	.078	0.931	(0.521–1.663)	.809

^a^Statistically significant. BMI, body mass index; UC, ureteral calculi.

### Sensitivity Analysis and Verification of Risk Factors(Subgroup Analysis)

After excluding nonsurgical patients, this study conducted a sensitivity analysis on 524 surgical patients, with the results detailed in Table 6. The results of this analysis further clarified that female gender, comorbid diabetes mellitus, and hydronephrosis size > 40 mm are independent risk factors for preoperative concurrent pathogenic pyuria in patients with UC.

**Table 6.** Risk Factors for Preoperative Concurrent Pathogenic Pyuria in Patients With UC(Subgroup Sensitivity Analysis)

Variables	Univariable			Multivariable		
	*OR*	*95% CI*	*P*	*OR*	*95% CI*	*P*
**Sex**						
Male	…	…		…	…	…
Female	5.111	(3.185–8.201)	<.001^a^	4.920	(2.950–8.204)	<.001^a^
**Age (y)**						
18–35	…	…		…	…	…
36–50	1.894	(0.862–4.159)	.112	1.557	(0.669–3.623)	.304
＞50	2.206	(1.041–4.675)	.039^a^	1.601	(0.700–3.661)	.265
**BMI**						
＜28	…	…		…	…	…
≥28	1.554	(0.800–3.020)	.193	1.852	(0.869–3.945)	.110
**Diabetes mellitus**						
NO	…	…		…	…	…
Yes	2.102	(1.074–4.115)	.030^a^	2.327	(1.076–5.034)	.032^a^
**Hypertension**						
NO	…	…		…	…	…
Yes	0.771	(0.463–1.283)	.317	0.543	(0.302–0.975)	.041^a^
**Concomitant kidney stones**						
No concomitant kidney stones	…	…		…	…	…
Concomitant ipsilateral kidney stones	1.320	(0.772–2.255)	.310	1.477	(0.811–2.690)	.202
Concomitant contralateral kidney stones	0.629	(0.318–1.244)	.182	0.660	(0.312–1.398)	.278
Concomitant bilateral kidney stones	0.791	(0.463–1.351)	.391	0.738	(0.407–1.339)	.318
**UC(unilateral/bilateral)**						
Unilateral UC	…	…		…	…	…
Bilateral UC	0.556	(0.290–1.066)	.077	0.712	(0.334–1.519)	.380
**Single/multiple stones**						
Single stone	…	…		…	…	…
Multiple stones	1.085	(0.639–1.843)	.762	1.007	(0.518–1.960)	.983
**Stone size(mm)**						
≤6	…	…		…	…	…
7–15	0.816	(0.463–1.436)	.480	0.847	(0.446–1.610)	.612
＞15	0.596	(0.271–1.311)	.198	0.562	(0.224–1.410)	.220
**Hydronephrosis size(mm)**						
≤10	…	…		…	…	…
11–20	3.064	(0.680–13.803)	.145	2.442	(0.497–12.008)	.272
21–30	3.559	(0.809–15.658)	.093	3.351	(0.703–15.964)	.129
31–40	5.035	(1.120–22.641)	.035^a^	4.450	(0.909–21.779)	.065
＞40	4.435	(0.970–20.284)	.055	6.574	(1.307–33.065)	.022^a^
**Stone location**						
Upper segment	…	…		…	…	…
Middle segment	1.222	(0.769–1.942)	.396	.814	(0.478–1.386)	.448
Lower segment	0.672	(0.224–2.018)	.478	0.764	(0.223–2.618)	.668
Multiple segment	0.962	(0.376–2.460)	.935	0.704	(0.215–2.309)	.562
**Whether renal function impairment**						
NO	…	…		…	…	…
Yes	0.570	(0.336–0.967)	.037	0.840	(0.451–1.565)	.583

^a^Statistically significant. BMI, body mass index; UC, ureteral calculi.

### The Inclusion Process of Predictive Variables

**Figure 1.**
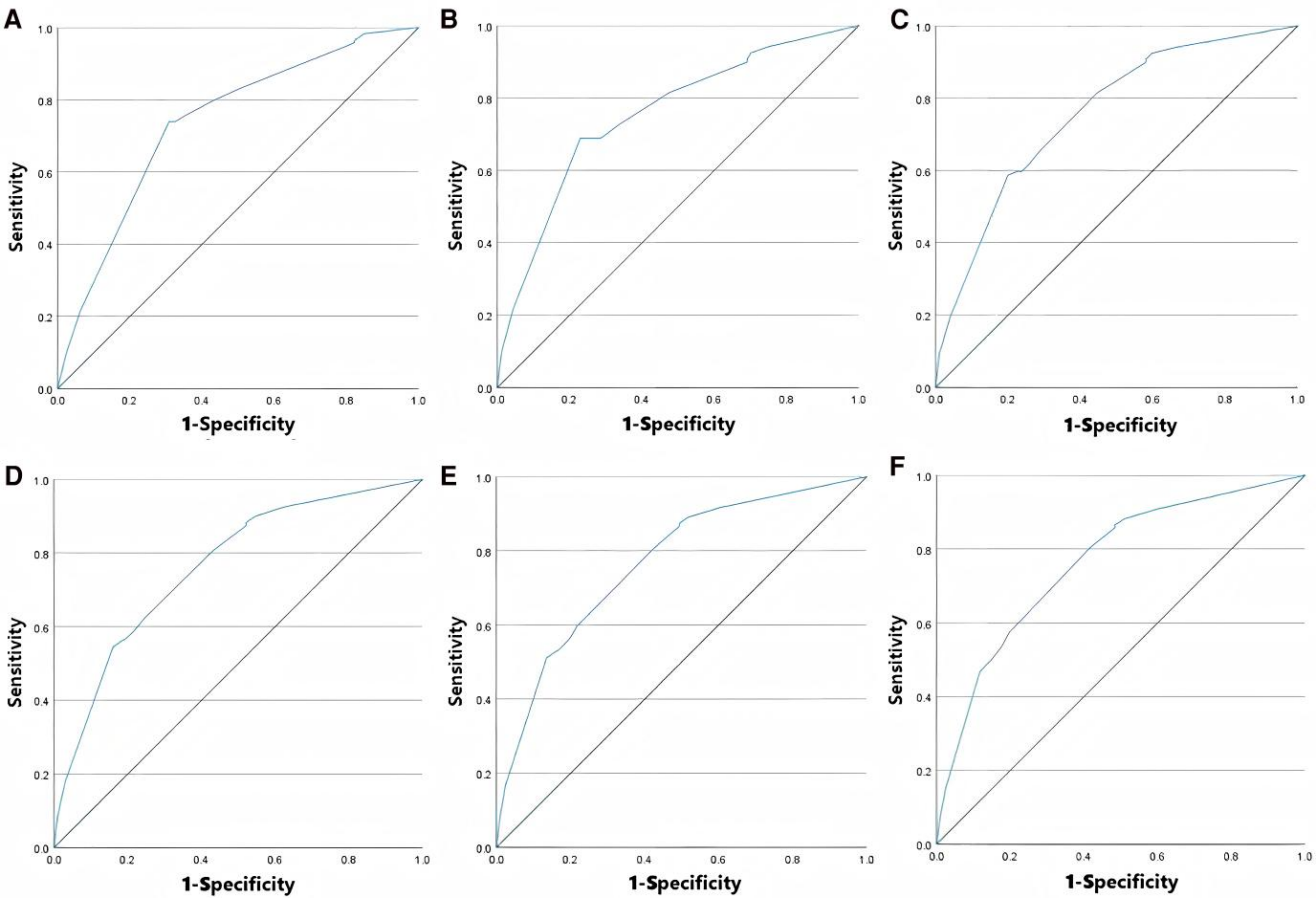
*A*, Receiver operating characteristic curve of the prediction model constructed by urinary WBC > 10/HPF combined with 3 other predictive variables (AUC = 0.738, H-L test *P* = .149); (*B*) urinary WBC > 20/HPF (AUC = 0.754, H-L test *P* = .034); (*C*) urinary WBC > 30/HPF (AUC = 0.756, H-L test *P* = .699); (*D*) urinary WBC > 40/HPF (AUC = 0.763, H-L test *P* = .778); (*E*) urinary WBC > 50/HPF (AUC = 0.766, H-L test *P* = .854); (*F*) urinary WBC > 60/HPF (AUC = 0.764, H-L test *P* = .989).

This study included 3 independent risk factors confirmed by subgroup sensitivity analysis as predictive variables for constructing the prediction model. Since the model needs to further predict the occurrence of concurrent pathogenic pyuria on the basis of confirmed pyuria, urine WBC count was also included as a predictive variable. To distinguish the impact of different urine WBC count levels on the prediction results, we divided them into 6 scenarios: urine WBC/HPF > 10, > 20, > 30, > 40, > 50, and > 60. For each scenario, the predicted probability was calculated via multivariate logistic regression analysis, ROC curves were plotted, and the Hosmer-Lemeshow test was performed (with *P*-value calculated). Based on the area under the ROC curve and the *P*-value of the Hosmer–Lemeshow test, the optimal classification criteria for constructing the prediction model were selected: when urine WBC > 60/HPF was included for joint model construction, the AUC was 0.764, the *P*-value of the Hosmer-Lemeshow test was 0.989, and the goodness of fit was the best. Therefore, it was included as a predictive variable (see Figure 1 for details).

### Construction and Validation of the Predictive Model

**Figure 2.**
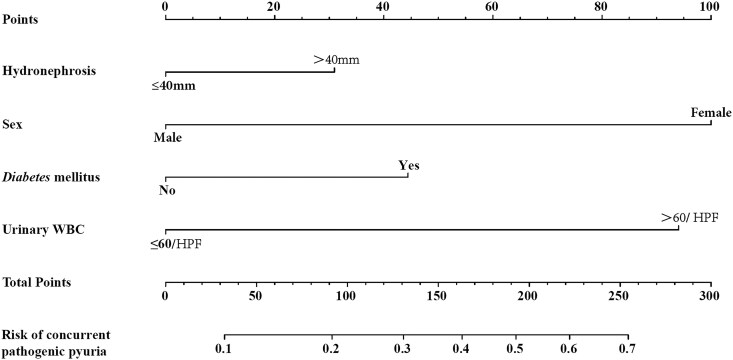
Nomogram for predicting the probability of preoperative concurrent pathogenic pyuria in patients with UC.

**Figure 3.**
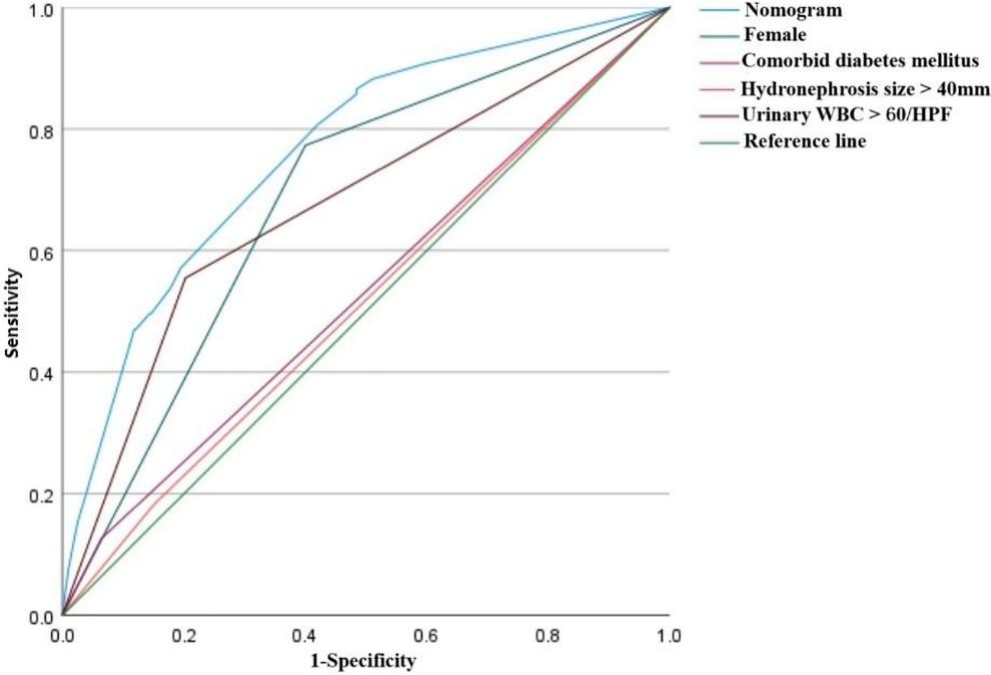
Comparison of ROC curves between the prediction model and individual indicators.

**Figure 4.**
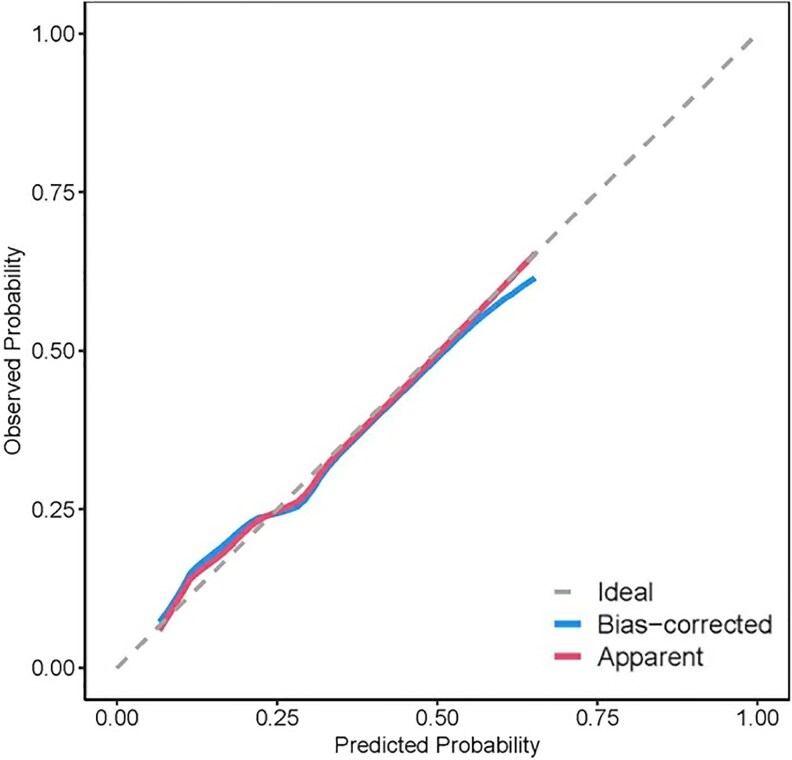
Calibration curve of the prediction model. The red line reflects the original performance of the model, and the blue line reflects the performance after internal validation using the Bootstrap method (B = 1000).

We selected female gender, comorbid diabetes mellitus, hydronephrosis size > 40 mm, and urine WBC > 60/HPF as predictive variables to construct a prediction model (Figure 2). The validation results are shown in Figure 3: the area under the ROC curve of this model is 0.764, which is superior to the application effect of individual risk factors, indicating that it has good discriminative ability; the *P*-value of the Hosmer–Lemeshow test is 0.989 (*P* > .05), suggesting that the model has a good degree of fit; after 1000 repeated internal validations with bootstrap samples, the calibration curve (Figure 4) shows that the predicted values of the model are highly consistent with the actual observed values.

## DISCUSSION

At present, the global prevalence of urolithiasis is showing an overall increasing trend year by year [18], which means that the number of urolithiasis patients requiring surgical treatment in clinical practice may also increase accordingly. Patients with urolithiasis are prone to concurrent UTIs [19]. Therefore, urinalysis in these patients often detects pyuria, with results available on the day of admission. In clinical practice, once pyuria is detected, clinicians will promptly initiate targeted anti-infective treatment based on the patient's specific condition, so pyuria generally does not affect the perioperative progress of urolithiasis patients. However, if preoperative urine culture indicates the presence of pathogenic bacterial infection, it is necessary to repeatedly recheck the urine culture after anti-infective treatment until the result is negative before surgery can be performed. Since urine culture results take at least 48 hours to be available, this not only delays the timing of early preoperative intervention but also significantly prolongs the duration of the patient's perioperative period. Studies have confirmed that pathogenic infection indicated by preoperative urine culture is closely associated with the occurrence of UTI after UC surgery [15]. Given the delay in urine culture results, clinical practice often sees delayed early preoperative intervention for patients with urinary pathogenic infections. This study aimed to explore the risk factors for preoperative pyuria in patients with UC. After confirming pyuria, it further analyzed the relevant risk factors for preoperative concurrent pathogenic pyuria in these patients and constructed a corresponding prediction model to assess the risk of preoperative concurrent pathogenic pyuria in this population earlier and more accurately. As confirmed by a search of the PubMed database, this study represents the first exploration into this issue. The results of this study showed that female gender, bilateral UC, stone size, and hydronephrosis size are risk factors for preoperative pyuria in such patients. Further analysis showed that the prediction model constructed based on female gender, comorbid diabetes mellitus, hydronephrosis size > 40 mm, and urine WBC >60/HPF can effectively predict the risk of preoperative concurrent pathogenic pyuria in female patients with UC. This helps clinicians identify such patients early and take timely intervention measures.

Pyuria, as a manifestation of UTI in urinalysis, is either used as an observation indicator or the focus of studies exploring risk factors for UTI in most existing research, while there is a near absence of research that directly focuses on the risk factors intrinsic to pyuria itself. However, in clinical practice, whether to initiate anti-infective treatment before surgery for patients with UC needs to be evaluated based on the WBC count in urinalysis and urine culture results. Currently, research on the risk factors intrinsic to pyuria itself is extremely scarce. Through a search in the PubMed database, we have not found any research literature specifically exploring the risk factors of pyuria itself. However, previous studies have confirmed that clinically significant UTIs are almost always accompanied by the presence of pyuria [20]. Yongzhi et al demonstrated that sex and multiple sites of stones act as independent factors of UTI in urolithiasis patients [21]. Sugizaki et al found that female gender is independent risk factors for urosepsis in patients with UC [22]. Additionally, some studies have also shown that being female gender and the size of stones act as independent risk factors for UTI [12, 23]. A study involving 599 patients with urolithiasis indicated that stone size is a risk factor for UTI in such patients [24]. He et al research revealed that stone size serves as an independent risk factor for UTI following surgery in patients with UC [25]. Hydronephrosis, as a direct manifestation of obstruction caused by UC, has been confirmed by studies to be significantly associated with an increased risk of UTI [26]. Wallis et al conducted a study on 299 neonates, and the results showed that the incidence of UTI was significantly higher in neonates with hydronephrosis [27]. Yet another study showed that in cases of obstructive uropathy, patients with severe hydronephrosis exhibit a 39% higher rate of UTI than those without obstruction [28]. Although different studies may yield varying conclusions, synthesizing the above research, we find that female gender, stone size, and hydronephrosis are all significantly associated with the occurrence of UTI [22–28]. The results of this study indicate that female gender, stone size, and hydronephrosis size are independent risk factors for preoperative pyuria in patients with UC. Different from previous studies focusing on the risk factors of UTI, this study did not directly explore the relevant risk factors of UTI, but analyzed the risk factors of pyuria associated with UTI. Despite the difference in research objects, the results of this study are similar to the conclusions of previous studies on the risk factors of UTI. Notably, this study also found that bilateral UC are another independent risk factor for preoperative pyuria in patients with UC. Among the 1182 subjects, there were 1056 patients with unilateral UC and 126 patients with bilateral UC, with the incidence rates of pyuria being 51.4% and 69.0% respectively. The difference between the 2 groups was statistically significant (*P* < .05). Subsequent multivariate logistic regression analysis and sensitivity analysis further verified this conclusion. Through a systematic literature search, no relevant reports with similar conclusions have been found to date. Therefore, it can be considered that this study is the first to confirm the conclusion that bilateral UC are an independent risk factor for preoperative pyuria in patients with UC.

Further analysis of the risk factors for preoperative concurrent pathogenic pyuria in patients with UC revealed that female gender is not only an independent risk factor for preoperative pyuria in such patients, but also an independent risk factor for their preoperative concurrent pathogenic pyuria. Rodríguez Del Águila et al's study indicated that there is a significant association between positive urine culture in adults and sex, with a higher positive rate in females. Among hospitalized adult patients, the risk of developing urinary bacterial infections in females is 36% higher than in males [29]. Similar research reports indicate that the probability of positive urine culture in females is significantly higher than in males [30]. Serretiello et al's study of 46 382 patients showed that 9896 had positive urine cultures, with 62.23% (6158 cases) being female and 37.77% (3738 cases) being male, suggesting a higher risk of urinary pathogenic infections in females [31]. Diabetes mellitus, as a chronic underlying disease, is more prone to bacterial UTIs due to impaired immune defense function and high glucose concentration in urine [32]. Geerlings et al included 636 diabetic women and 153 nondiabetic women as controls in their study. The results showed that the positive rate of urine culture was 26% in the diabetic group and only 6% in the control group, with a statistically significant difference [33]. A similar study by Laway et al showed that the prevalence of asymptomatic bacteriuria in patients with type 2 diabetes (17.5%) was significantly higher than that in the control group (10%), with *P* = .015, suggesting that the prevalence in diabetic patients is significantly higher [34]. This study found that comorbid diabetes mellitus is an independent risk factor for preoperative concurrent pathogenic pyuria in patients with UC, that is, those with comorbid diabetes mellitus have a higher risk of such infections than nondiabetic mellitus patients, which is consistent with previous research conclusions. Our study also confirmed that hydronephrosis size >40 mm is an independent risk factor for preoperative concurrent pathogenic pyuria in patients with UC. Sgayer et al conducted a study on 262 pregnant women, and the results showed that hydronephrosis was significantly associated with the occurrence of repeated positive urine cultures [35]. Kitano et al included 286 patients with staphylococcal aureus bacteriuria in their study, and the results indicated that there is a significant correlation between hydronephrosis and UTIs associated with Staphylococcus aureus [36]. A retrospective analysis of 1000 patients who underwent surgical treatment by Gofrit et al showed a significant correlation between hydronephrosis grading and positive preoperative urine culture [37]. Our research results not only validate the conclusion that there is a significant correlation between hydronephrosis and positive urine culture, but also further confirm that in patients with UC, when the size of hydronephrosis exceeds 40 mm, the risk of concurrent pathogenic pyuria before surgery will significantly increase. This study confirms that female gender, comorbid diabetes mellitus, and hydronephrosis size > 40 mm are independent risk factors for preoperative concurrent pathogenic pyuria in patients with UC. On this basis, we further constructed a predictive model by taking the above 3 indicators together with urinary WBC > 60/HPF as predictive variables. This model is capable of effectively forecasting the risk of preoperative concurrent pathogenic pyuria in female UC patients, thereby offering clinicians a dependable reference for carrying out early preoperative interventions in this patient group.

This study has certain limitations: first, given that this is a retrospective study, it is inevitably prone to selection bias; second, the sample size of the subgroup analysis is small and the included indicators are not comprehensive enough, which may affect the efficacy of the prediction model; third, the constructed prediction model is only applicable to risk assessment in female patients with UC and cannot be generalized to male patients; fourth, due to the relatively small amount of data in the subgroup analysis, only internal validation of the model has been completed so far, with a lack of external validation. Therefore, it is necessary to expand the sample size for further validation.

## CONCLUSIONS

This study focuses on exploring the risk factors associated with preoperative pyuria and concurrent pathogenic pyuria in patients with UC, a research direction that has not been reported similarly in previous literature. The results show that female gender, bilateral UC, stone size, and hydronephrosis size are independent risk factors for preoperative pyuria in such patients; further research confirms that female gender, comorbid diabetes mellitus, and hydronephrosis size > 40 mm are independent risk factors for their preoperative concurrent pathogenic pyuria. Based on the above research findings, the predictive model we constructed can effectively predict the risk of preoperative concurrent pathogenic pyuria in female patients with UC, providing a reliable basis for clinicians to implement early preoperative intervention in such patients.
